# Conference Report

**Published:** 1964-04

**Authors:** Thomas S. Wilson

**Affiliations:** Consultant Geriatrician, Cornwall


### CONFERENCE REPORT

### SIXTH INTERNATIONAL CONGRESS OF GERONTOLOGY

The Congress in Copenhagen was attended by almost 1,000 delegates from 35 different countries.

An interesting series of papers dealt with the hereditary and environmental factors concerned with ageing. A report from Denmark comparing the ages at death of a large series of identical and non-identical twins emphasised the importance of inheritance in determining the life span. Demographic studies from a number of countries, including Russia and Rumania, dealt with environmental factors in relation to longevity. It was difficult to draw conclusions from these and indeed it was evident that these relationships are only vaguely understood. Improvements in standards of living, social circumstances, hygiene and advances in medical science are all concerned in the great numbers of the populations who survive till later life. In spite of these, however, the expectation of life of a person at the age of 60 years has not changed significantly in the past 50 years.

Perhaps one conclusion from the Institute of Geriatrics at Bucharest, following a survey of 30,000 patients, is worth quoting:—

"It is pointed out that most cases of premature ageing occurred either in over-strained intellectuals or in people leading an irregular way of life."

There were papers of particular interest on the Clinical Section, and I should like to mention two of these.

(1) A study was presented from Houston, Texas, of more than 500 patients over 65 years of age requiring vascular surgical procedures for aneurysms and occlusive lesions produced by arteriosclerosis. The indications and contra-indications for surgery, and the results (including long-term survival) were discussed. It is evident that this is an expanding field which may be of great importance in the future.

(2) A description of recent experimental and clinical work in the use of rheography was given by Ziemnowicz (Washington). This is essentially a method of assessing circulation changes in the brain by means of the variation in potential between electrodes on the scalp. Evidence was presented to suggest that this is a reliable and sensitive method of localising circulatory disorders in the brain, and that the anterior and middle cerebral arteries and the vertebral-basilar system can be selectively studied. The method has several advantages over angiography in that it permits continuous study of blood flow over a period, and is without risk or discomfort to the patient.

While in Copenhagen we visited several hospitals in the city:—

(1) *De Camles By*. (*Old Peoples' Town*)

The "Old Peoples' Town" in Copenhagen is an institution dating back to a time when social welfare was quite different in character from today. At present some 1,800 old people live there. The Danes freely admit that one would hardly opt for concentrating so many of them in one place if a similar institution were to be planned today. It was opened in the late 19th century, and in its day was an extremely progressive example of social welfare, in that special provision was made for the requirements of old people, in an age when, in this country, old people were mainly catered for in workhouses, together with all other types of socially destitute.

(2) *New General Hospital, Glostrop*

This is a new general hospital of 800 beds which was completed in 1958.

Lavishness in space is evidently a feature of the planning of Scandanavian hospitals, and the ward units consist mainly of single and double bedded units, which make for some difficulty in nursing. One felt on the whole that the building was somewhat impersonal, and one did not get the same impression of activity that is evident in a busy general hospital in this country. Perhaps this was accounted for in part by the fact that in Denmark there are no hospital out-patients.

One excellent feature was the provision in the planning for several shops in the main foyer similar to those seen in the foyer of large hotels. These are taken over by commercial concerns which are responsible for running them. They provide an important amenity for patients and visitors, and in particular the florist's shop ensures that there is a regular supply of flowers to the wards, being situated where the majority of visitors wait before going to see their friends.

THOMAS S. WILSON,

*Consultant Geriatrician, Cornwall.*

